# Recovery of Non-Ferrous Metals from PCBs Scrap by Liquation from Lead

**DOI:** 10.3390/ma15062089

**Published:** 2022-03-11

**Authors:** Maciej Wędrychowicz, Andrzej Piotrowicz, Tomasz Skrzekut, Piotr Noga, Adam Bydalek

**Affiliations:** 1 Institute of Materials and Biomedical Engineering, Faculty of Mechanical Engineering, University of Zielona Gora, 65-516 Zielona Gora, Poland; abydalek@uz.zgora.pl; 2 Faculty of Non-Ferrous Metals, AGH University of Science and Technology, 30-059 Krakow, Poland; andpio@agh.edu.pl (A.P.); skrzekut@agh.edu.pl (T.S.); pionoga@agh.edu.pl (P.N.)

**Keywords:** PCB recycling, high-temperature process, lead, liquation

## Abstract

This article presents the results of research on the recycling of non-ferrous metals from PCB scrap using melting in metallic lead. The idea of this process is to dissolve (transfer) metals from PCB scrap in lead, and then liquation them by cooling the lead-metals alloy. PCB scrap was crushed and then melted into liquid lead. The lead after process was then poured into the casting mold and its chemical composition was examined. Among the various metals in the PCB scrap, copper and tin in particular are dissolved in lead. The more scrap dissolved in lead, the higher the concentration of copper and tin in the alloy. The highest obtained concentration of copper in lead were about 2.2 wt.%, and for tin about 0.8 wt.%.

## 1. Introduction

The methods of recycling non-ferrous metals are conventionally divided into hydro- and pyrometallurgical methods [1,2], often preceded by mechanical processing [3,4]. Each of them has its advantages and disadvantages. Hydrometallurgical recycling requires the use of various types of acid and/or alkaline solutions, the amount and concentration of which depend on the type of scrap and the intermediate stages of processing. The most important disadvantages of hydrometallurgy include: the use of reagents that have a negative impact on health and the environment (e.g., HCl, HNO_3_), a high ratio of the volume of solutions to the solid phase (which is particularly important in the case of scaling processes towards industrial processes), the need to utilize waste solutions, etc. The most important advantage of hydrometallurgy is the possibility of selecting the technological parameters of the stages in such a way as to obtain high recovery efficiency of individual metals, in other word-selectivity. This is, of course, connected with a fairly large number of these steps to separate each metal and with the resulting waste solutions [5,6]. Pyrometallurgical recycling is based on the use of high temperature, i.e., separation due to the melting/boiling point of individual components and/or their solubility depending on the temperature. The disadvantages of pyrometallurgy include: the requirement to use high temperature (often above 1000 °C) and the associated emission of pollutants, such as volatile organic compounds, tar, and ash. An undoubted advantage of pyrometallurgical processes is scalability—the larger the scale of processing, the more profitable it is. The yields obtained by pyrometallurgical method are strongly dependent on such physicochemical phenomena as viscosity or surface tension, hence it is more difficult to obtain pure metals and almost always, additional hydrometallurgical treatment is required. Nevertheless, pyrometallurgy leads to rich metal concentrates that are easy to further process [7,8,9,10].

To compare the hydro- and pyrometallurgical treatments of PCB scrap, we used the following examples. Hydrometallurgy requires that the metallic components of electronic scrap be oxidized, because only in this form can they be leached or their leaching is technologically easier. In electronic scrap, metals such as copper, gold and silver are in a metallic form, and their oxidation is associated with the loss of energy and their raw material potential (i.e., loss of energy that was previously used to reduce the oxidized form in the concentrate; metallic components are more valuable than their oxidized forms) [9,11]. For this reason, for example, the recycling of brass is not carried out by hydrometallurgical means, but by pyrometallurgy. Recycling of brass is the same as the melting of scrap/concentrate to produce recycled brass [12,13].

The processing of electronic scrap is practiced, for example, in copper metallurgy. It is known that copper is a collector of precious and platinum group metals [14,15,16]. Various types of scrap, including metals such as copper, silver, gold, and platinum group metals, e.g., catalysts, electric/electronic scrap, are melted in the converter furnace [17]. By melting them, the valuable metallic components dissolve in the copper and eventually form the copper blister. The blister copper hydrometallurgical processing enables the recovery of these metals from the anode sludge [18,19].

**Figure 1:**
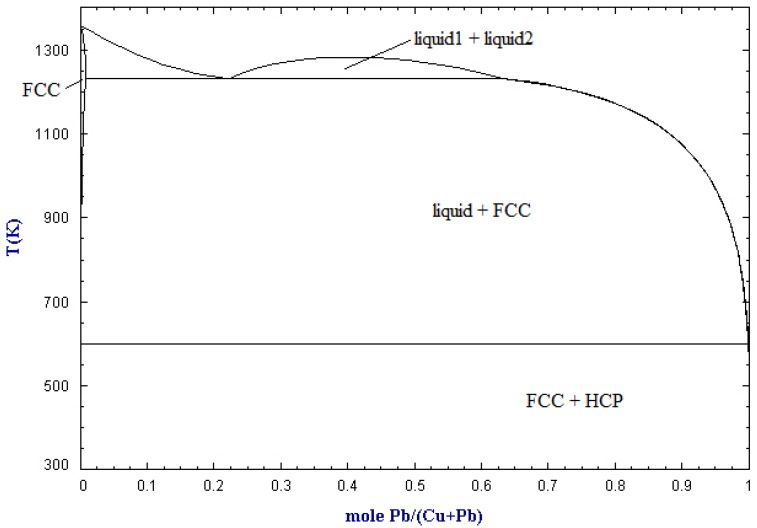
Cu-Pb phase diagram (based on [20]).

**Figure 2:**
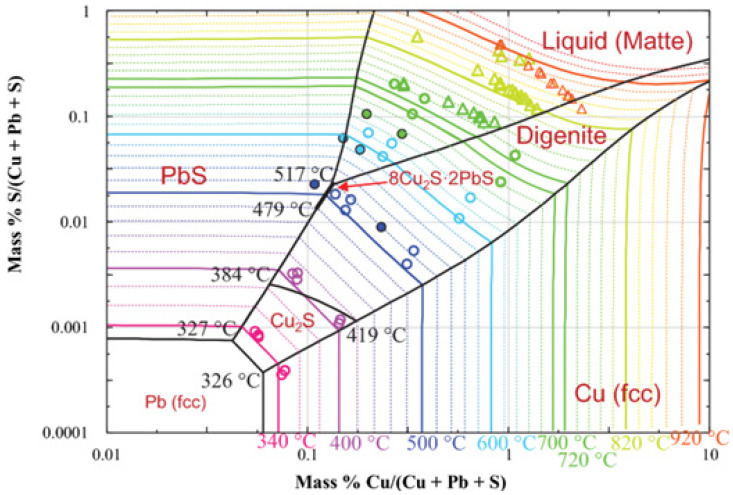
Cu-Pb-S phase diagram [21].

The solubility of copper in lead in molar ratio and temperature functions is shown in Figure 1 [20]. Above approx. 950 °C, there is only a liquid phase in the Cu-Pb system, which means that copper is completely dissolved in lead. In the range of low concentrations of copper in the system (mole fraction close to 1), as the temperature decreases along the liquidus line, the amount of the liquid phase decreases to the temperature of approximately 330 °C, at which there is practically no liquid phase left; it is thus a eutectic system with no miscibility (solubility) between Pb and Cu in the solid phase. This phenomenon is used practically in the refining of lead from copper in the liquation process. Crude lead, containing a few percent by mass of copper, is melted at about 500 °C, and then it is spontaneously slowly cooled down to a temperature of about 350 °C [21]. During cooling down, the so-called copper drosses, constituting the separated copper. An alternative or subsequent step is refining by using sulfur/lead sulfide (Figure 2). This practically involves adding sulfur (and/or sulfur compounds like iron sulfide) to the molten crude lead. Sulfur first reacts with lead (due to activity) and then the lead sulfide reacts with the copper to form copper sulfide, which accumulates on top of the melt [22]. Another method to refining copper (and other metals) from crude lead is using metallic aluminum, which makes a eutectic alloy of aluminum and impurities mechanically easy to remove from lead [23,24]. Both types of copper drosses, metallic copper and copper sulfide, are a by-products of lead refining and a copper concentrate directed to the next processing stages [25,26,27,28,29,30].

The use of lead instead of copper as a collector of precious metals (including copper in the case of lead) may be technologically more advantageous for several reasons. Lead has a lower heat of melting than copper (25 kJ∙kg^−1^ (Pb) vs. 205 kJ∙kg^−1^ (Cu)). The use of lead as a collector instead of copper is expected to result in higher dissolution efficiencies due to the lower viscosity of lead than copper (0.95 mPs∙s (Pb, 1114 °C [31]) vs. 4.03 mPa∙s (Cu, 1083 °C [32]). Practically, both collectors can be used under oxidative conditions because the free enthalpy values of their oxidation reaction are quite high (but still negative; −88 kJ∙mol^−1^ (Pb, 1100 °C) vs. −35.3 kJ∙mol^−1^ (Cu, 1100 °C) [33]. Overall, lead has always been cheaper than copper, which is of economic benefit. Electronic scrap melting can be applied in the lead production process (lead refining by liquation), similar to copper metallurgy (copper melting during converter process). Lead melting can provide an alternative to copper melting, especially in those places where copper metallurgy is absent. The recovery of precious and platinum group metals in copper metallurgy results from hydrometallurgical refining of copper, while their recovery in lead metallurgy can only be by pyrometallurgical route.

The aim of the research was to carry out the proposed method of recycling non-ferrous metals from PCB scrap as a result of melting in liquid lead. The chemical composition of various PCB scraps and the influence of the ratio of the amount of PCB scrap to crude lead on the amount of dissolved metals in liquid lead were investigated. For this purpose, the chemical composition was tested using the ED-XRF method. To confirm the change in the chemical composition on a microscopic scale, element mapping of the post-process lead was also performed.

## 2. Materials and Methods

**Figure 3:**
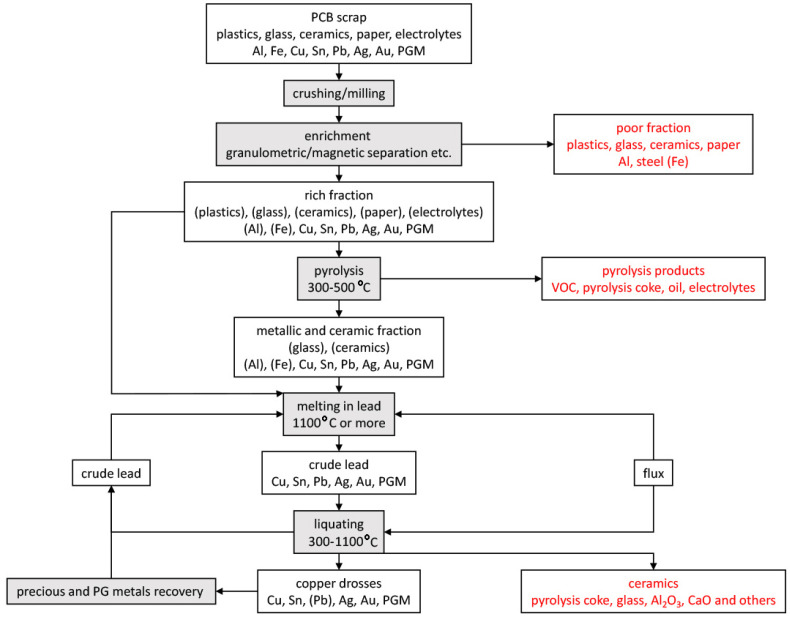
Scheme of non-ferrous metals recovery process from electrical/electronic scrap using lead melting and liquation.

The presented idea concerns the recycling of non-ferrous metals from electronic scrap by pyrometallurgical route. It is based on the solubility of the non-ferrous metals in electronic scrap in lead, followed by liquation to form a concentrate of copper and accompanying precious metals. The processing of electronic scrap with the proposed method is shown in Figure 3 (those components whose content should be residual are given in brackets). First, the electrical scrap is pre-treated by known methods such as grinding, particle size, or magnetic separation in order to separate waste components (plastics, ceramics, etc.) from metals. Then, the so-called rich fraction is subjected to pyrolysis in order to remove volatile components (plastics) and electrolyte (included in batteries and capacitors). The pyrolysis products can be further processed to recover oils and pyrolysis coke. The rich fraction can also be sent directly to the lead melting stage as the melting in lead takes place at a temperature higher than the pyrolysis. Lead melting should take place at a temperature of at least 1100 °C, so that the liquid phase is the predominant phase (see Figure 1). By melting, copper and other accompanying metals are dissolved in lead. Both pure lead and crude lead can be used for this. As a result of liquation, the metallic components dissolved in the lead precipitate in the form of drosses. The liquation is carried out at a temperature below the melting, preferably up to 350 °C. A flux can be used in both the melting and liquating steps to lower the melt viscosity. The use of flux is particularly advantageous in the liquation stage when lead becomes sticky at lower temperatures. The flux in the liquation step may be, for example, paraffin. Crude lead is a by-product of the liquation process, which can be re-directed to the next melting of electronic scrap. As a result of the correct mechanical separation of the drosses from the top (carried out e.g., with a perforated colander type tool), it should be possible to mechanically separate the lead, drosses, and the part undissolved at the melting stage, i.e., various types of ceramic components and aluminum skimmings. The copper drosses can then be further processed in successive leaching steps (which further recovers crude lead and concentrates copper and other accompanying metals) or by a hydrometallurgical process.

The aim of the laboratory tests was to investigate whether the method of non-ferrous metals recycling from PCB scraps by the proposed method is possible and effective. PCB scraps with the following operational markings: “B1”, “B3”, “B5”, “server”, “new”, were initially enriched and then pyrolyzed (“B1”—boards from xero devices, printer, fax, without heavy elements such as transformers, large capacitors, heat sinks, include gold-plating sockets; “B3”—PCBs from xero devices, printer, fax, with heavy elements such as transformers, large capacitors, heat sinks, little gold plating; “B5”—boards with xero devices, printer, fax, router, modem–mix; “server”—server board backplane, bulkhead walls; “new”—server board with two sockets, new scrap). These pyrolysate were melted in lead. The compositions of crude lead and post-processing lead (after melting with PCB scrap) were determined in order to verify whether the metal components had transferred into the lead. From a research point of view, it was important to investigate how much PCB pyrolysate could be melted with crude lead, which was also investigated. The chemical compositions of PCB scraps, crude lead, and post-process lead were investigated by the EDXRF method (ED-XRF, Minipal 4, PANalytical).

### 2.1. PCB Scrap Enrichment

PCB scraps were grinded on a shredder, and then the fine fraction was separated from the coarse fraction using the wet method (water and detergent). A fine fraction, consisting primarily of glass and fine aluminum, rose to the top of the aqueous bath, and the coarse fraction fell to the bottom. The fine fraction was rejected as a poor fraction unsuitable for melting in lead.

### 2.2. Pyrolysis

Coarse fraction of PCB scraps from enrichment step, called the initial material, was placed in tall and narrow alumina crucibles (D = 20 mm, H = 200 mm) and subjected to pyrolysis in an electric furnace under an argon atmosphere. The temperature and duration of the pyrolysis were 500 °C and 1 h for each operational markings material, respectively. As a result of pyrolysis, volatile components such as plastics and foils are removed. The weight loss as a result of pyrolysis was calculated on the basis of the Equation:

(1) $$∆m=\frac{m_{0}-m_{1}}{m_{0}}\cdot 100\%$$

where: ∆*m* is weight loss resulting of pyrolysis, *m*_0_ is mass of the initial material (before pyrolysis), and *m*_1_ is the final mass of the pyrolysate (after pyrolysis). The higher the weight loss value, the more the pyrolysis gangue components were removed.

### 2.3. PCB Pyrolyzate Melting in Lead

Technical-grade lead for melting with PCB scrap was firstly refined by the Harris method (electric furnace, steel crucible, steel mold, 500 °C, 60 min, mixing the molten mixture every 20 min) maintaining the following proportions of refining salts per one kilogram of lead: 22.8 g NaNO_3_, 70.2 g NaOH, 23.4 g NaCl.

Attempts to melt PCB scrap pyrolysate with crude lead were as follows:

1. lead ingot and scrap pyrolysate were placed in a graphite crucible;
2. graphite crucible with its contents was placed in an alumina crucible;
3. graphite crucible was covered with a graphite lid;
4. free space between the crucibles and the top part of lid was fulfilled with alumina powder;
5. whole was placed in an electric furnace and;
6. heated together with the furnace to a preset temperature.

**Figure 4:**
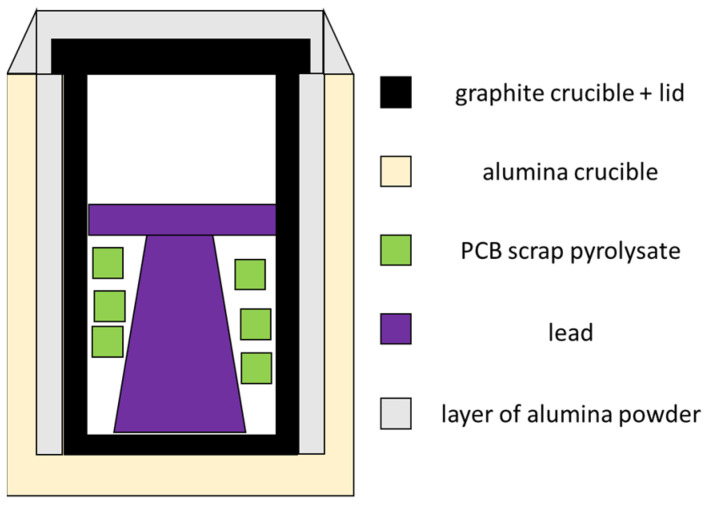
Scheme of the system cross-section of materials before PCB scrap melting with lead.

A scheme of the system cross-section is shown in Figure 4. Due to the corrosive nature of lead, lead was melted in a graphite crucible (otherwise, the alumina crucible would corrode by diffuse liquid lead). Graphite was protected from an oxidizing atmosphere by placing a graphite crucible in an alumina crucible; alumina powder provided additional protection against excessive graphite combustion. The whole system was heated with the furnace from room temperature to the temperature of the process, i.e., 1100 °C, and kept there for 1 h. The crucible was opened one time and the contents were mixed with a heated metal rod with a perforated sheet to homogenize the top, and then the crucible was again covered with the lid. The variable parameters for melting PCB scrap in lead were the batch of the scrap (out of five markings available) and the ratio of the mass of scrap to the mass of the crude lead: 13, 26, and 40 g of scrap per 400 g of crude lead (approximate values, depending on the mass of the crude lead). From the obtained ingots after the melting process, three pieces (from the bottom, center, and top) were cut, ground and analyzed for chemical composition. Chemical composition of lead after melting process was also examined using the scanning electron microscope with the energy dispersion spectroscopy system (SEM-EDS) (SU-70, Hitachi, Chiyoda City, Tokyo, Japan) as well as element mapping were performed.

## 3. Results and Discussion

### 3.1. Enrichment Steps of PCB Scraps

The composition of the initial material, i.e., PCB scrap after the enrichment step, is presented in Table 1 (elements composition as oxides). The initial material consists mainly of aluminum and SiO_2_ (59–83%), some CaO (8–14%) and copper (2–10%). Bromine content results from the composition of printed circuit boards (bromine increases the flash point of the board, which is necessary in the process of soldering components), and tin and lead from the composition of the solder. Probably due to too low concentrations, silver, gold, and platinum group metals were not registered. Out of the initial materials, “B3” seems to be the worst scrap, from a material point of view, due to its high aluminum and silica contents as well as its low copper concentration (only 2% compared to the rest 7–10%). The composition of these PCB scraps are similar to that presented in [34]. The analysis does not take into account the carbon content of plastics, hence the results of the analyzes are arbitrary and serve only to compare the composition of scrap with each other.

The calculated values of weight loss resulting of pyrolysis are presented in Table 2. Apart from “B3”, the values of losses for the remaining materials are 26–37%; for “B3” the value of the weight loss is almost 83%. The high value of the loss and the low concentration of copper in it indicate that the “B3” enrichment did not proceed as correctly as the other materials. This may also be because material “B3” is poor in metals and rich in pyrolysable components. Nevertheless, at least 25% of the starting weight was removed by pyrolysis. The material after pyrolysis was dark, charred, brittle, and it was easy to distinguish metallic components, especially metallic copper.

### 3.2. PCB Pyrylozates Melting with Lead

**Figure 5:**
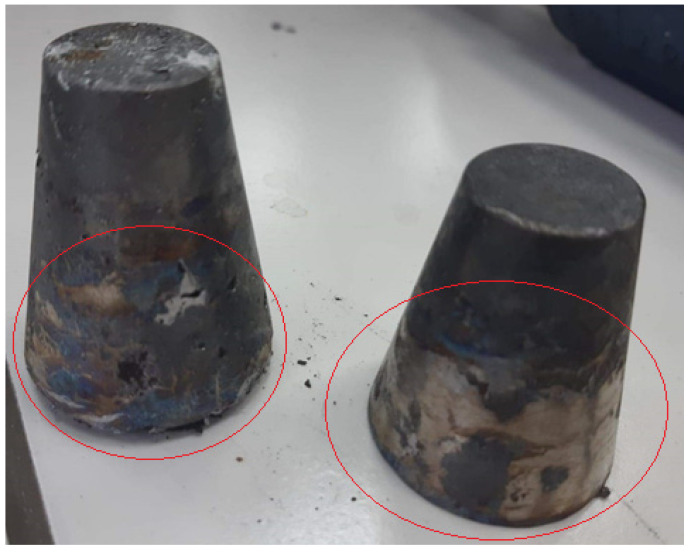
Appearances of ingots after pouring molten lead with PCB, red circles show a colorization of ingots.

The averaged chemical composition of crude lead used for melting PCB pyrolyzates are presented in Table 3 (a piece was cut from the whole ingot, ground and analyzed, average of ten different ingots). Compared to crude lead, lead after melting the PCB pyrolyzate had a brown-golden and purple color (Figure 5), which may indicate an increased concentration of non-lead metals as copper (purple) and/or tin (brown-golden). The colors of the ingots appear only on the upper part of the ingots, which proves that drosses are formed when the alloy cools down to the appropriate temperature and that they flow to the top (due to their lower density and surface tension). The chemical compositions of ingots after process are presented in Table 4, Table 5, Table 6, Table 7 and Table 8. The presence of calcium in crude and post-process lead ingots is due to primary contamination. The calcium concentration in lead after the process was in some cases higher than the calcium concentration in the crude lead, i.e., up to 0.389 wt.% vs. 0.284 wt.%. The presence of iron and nickel has the same genesis as calcium, and likewise with calcium, it does not matter much. It shows, however, that the ferrous metals that were present in the initial material (in the amount of 0.97–4.98 wt.% Fe_2_O_3_ and 0.589–0.749 wt.% NiO) do not dissolve in lead, because otherwise their increased concentration in lead would be noted after the process compared to crude lead (0.042 wt.% and 0.502 wt.% Fe and Ni vs. 0.275 wt.% and 0.667 wt.% Fe and Ni, respectively for crude lead and lead after the process). In the cases of melting of “B3” and “B5” pyrolysates, a small gold content was also noted in lead ingots after melting, in the amount of 0.04–0.1 wt.%. Overall, an increase in the concentration of metals such as copper and tin can be seen compared to the crude lead (compare Table 3, Table 4, Table 5, Table 6, Table 7 and Table 8). Depending on the initial material and the ratio of the weight of scrap to the weight of lead (*m_PCB_*/*m_Pb_*), the concentrations of copper and tin were 0.5–3.2 and 0.4–1.3 wt.%, respectively. This proves that copper and tin are dissolved in lead. The metal contents in lead depend not only on the *m_PCB_*/*m_Pb_* ratio, but also on the initial material. Thus, the smallest amount of tin was after melting “B5” at *m_PCB_*/*m_Pb_* = 0.0325 g∙g^−1^, i.e., 0.429 wt.%, and the most after melting “new” at *m_PCB_*/*m_Pb_* = 0.1 g∙g^−1^, i.e., 1.358 wt. %; in the case of copper: the smallest—after melting “B3” at *m_PCB_*/*m_Pb_* = 0.0325 g∙g^−1^, i.e., 0.538 wt. %, and the most, as much as 3.206 wt.%, after melting “new” at *m_PCB_*/*m_Pb_* = 0.1 g∙g^−1^. Overall, it appears that the liquation of tin and copper after melting of pyrolysate “new” in lead will be most effective due to the relatively high concentrations of copper and tin.

**Figure 6:**
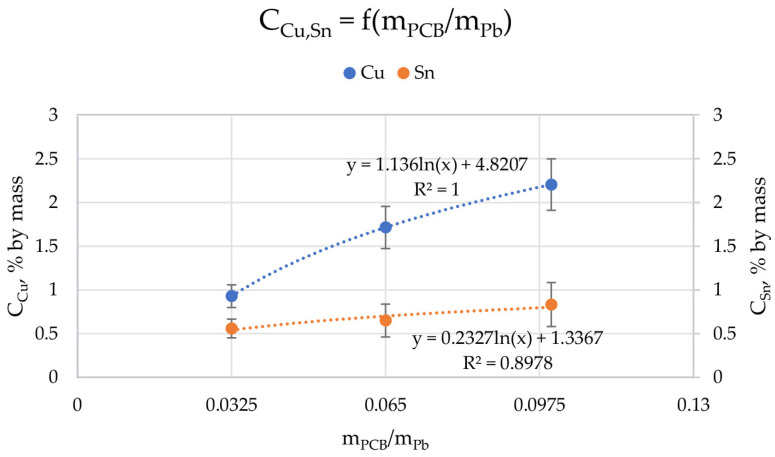
Relationship between the ratio of the weight of scrap to the weight of lead (*m_PCB_*/*m_Pb_*) and copper (C_Cu_) and tin (C_Sn_) concentrations in ingots after process.

Figure 6 shows the relationship of the concentration of copper and tin on *m_PCB_*/*m_Pb_* (the values of Cu and Sn concentrations in all ingots were averaged for individual *m_PCB_*/*m_Pb_*). It was to be expected that the higher the proportion of PCBs in lead (the higher the *m_PCB_*/*m_Pb_* value), the more copper and tin should be in the lead due to their solubility, which turned out to be true. We can talk about a logarithmic relationship of the concentration of Cu and Sn on *m_PCB_*/*m_Pb_*. The *m_PCB_*/*m_Pb_* has a greater influence on the amount of copper than tin in lead (1.126 · ln (*m_PCB_*/*m_Pb_*) + 4.8207 vs. 0.2327 · ln (*m_PCB_*/*m_Pb_*) + 1.2267, for copper and tin, respectively). However, this relationship is expected to be non-linear across the mass ratio range due to the limited solubility of copper and tin in lead. It should be remembered that due to the solubility of these metals in lead and the process conditions, mainly temperature, the amount of PCB that can be melted with lead is limited. Another limiting factor is the mere physical presence of PCB scrap, the more of which there are, the more difficult it can be to mix the top and cause splash losses.

**Figure 7:**
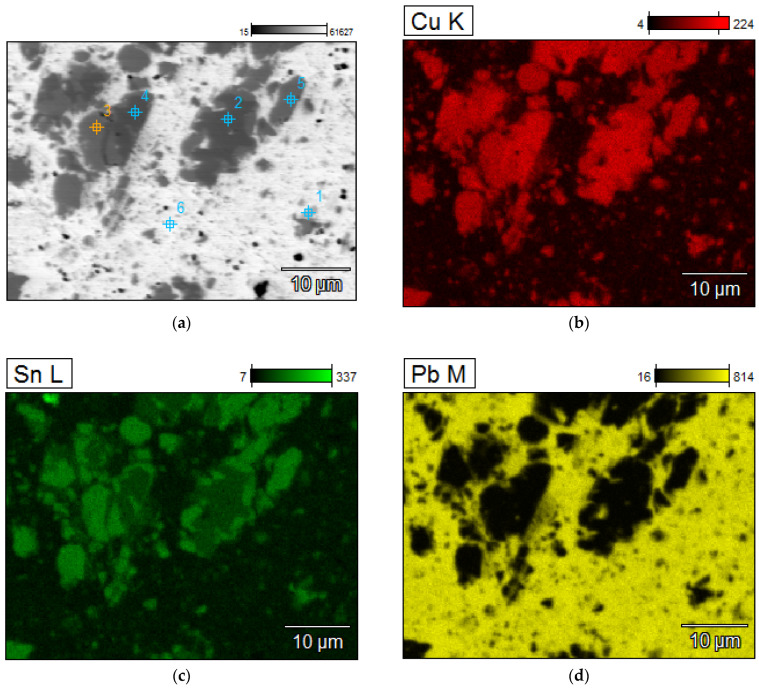
Elements mapping of the lead ingots after melting with PCB pyrolyzate “B1”, *m_PCB_*/*m_Pb_* = 0.1 g∙g^−1^. (**a**) scanning electron microscopy with a points of chemical composition; (**b**–**d**) copper, tin and lead located in specific areas within the alloy.

**Figure 8:**
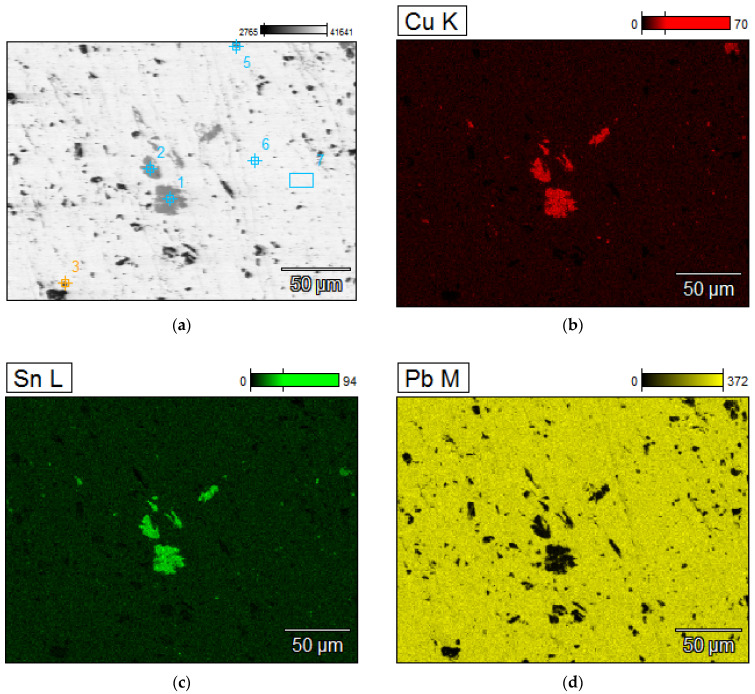
Elements mapping of the lead ingots after melting with PCB pyrolyzate “B3”, *m_PCB_*/*m_Pb_* = 0.1 g∙g^−1^. (**a**) scanning electron microscopy with a points of chemical composition; (**b**–**d**) copper, tin and lead located in specific areas within the alloy.

**Figure 9:**
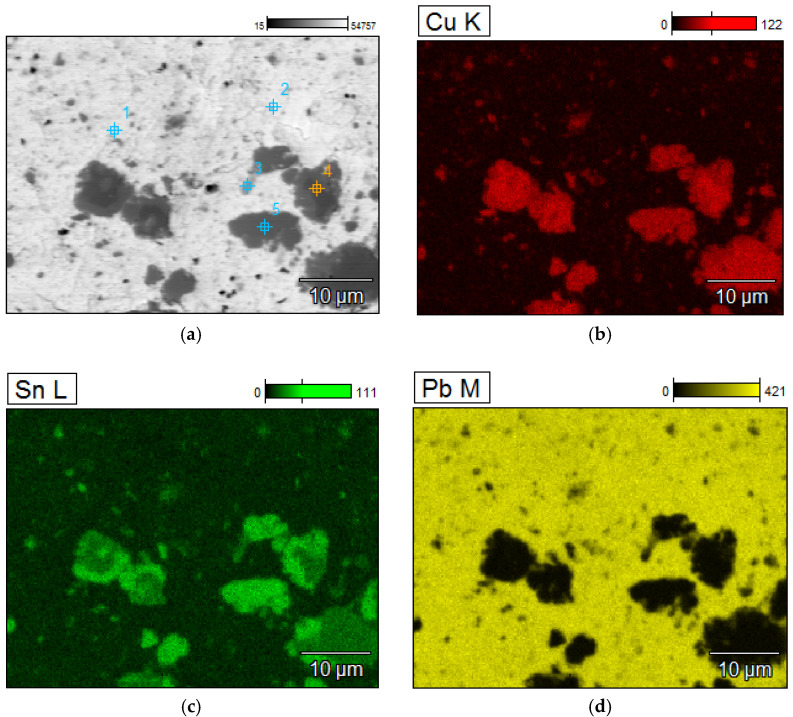
Elements mapping of the lead ingots after melting with PCB pyrolyzate “server”, *m_PCB_*/*m_Pb_* = 0.1 g∙g^−1^. (**a**) scanning electron microscopy with a points of chemical composition; (**b**–**d**) copper, tin and lead located in specific areas within the alloy.

**Figure 10:**
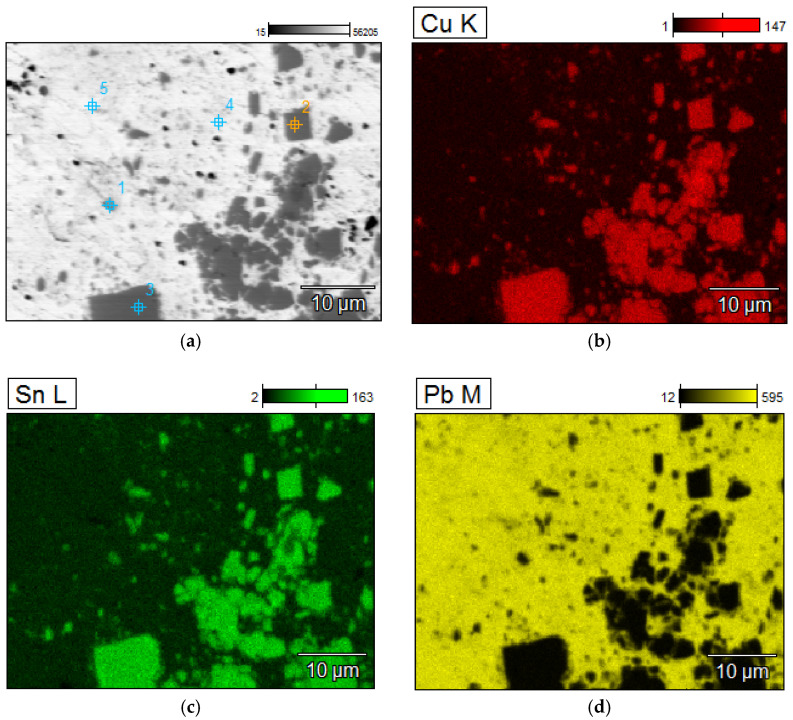
Elements mapping of the lead ingots after melting with PCB pyrolyzate “new”, *m_PCB_*/*m_Pb_* = 0.1 g∙g^−1^. (**a**) scanning electron microscopy with a points of chemical composition; (**b**–**d**) copper, tin and lead located in specific areas within the alloy.

A detailed analyzes of the ingots are shown by microscopic photos, elements mapping (Figure 7, Figure 8, Figure 9 and Figure 10, for “B1”, “B3”, “server”, and “new”, respectively, all for *m_PCB_*/*m_Pb_* = 0.1) and point and area analyzes (Table 9, Table 10, Table 11 and Table 12, for “B1”, “B3”, “server”, and “new”, respectively, all for *m_PCB_*/*m_Pb_* = 0.1). In all cases, it is easy to notice that the lead is rich in copper and tin (bronze) phases, where the concentration of copper reaches 89%, and tin 26%. These bronzes contain almost no lead and as such could precipitate out as a result of liquation. These phases are of small size (from a few to several dozen microns), which is additional evidence that these phases must have precipitated from cooling lead and not be present in the initial material. Apart from bronzes, there are also less frequent phases rich in silver (up to 88% by mass). Silver can be recovered in the form of the so-called silver-bearing foam in the stage of refining lead from zinc (examples in [35,36]). Only one phase containing a significant amount of Al was recorded (up to 22%, point 5 of sample “B3”) and this is probably an inclusion resulting from casting to mold. In general, the concentration of Al in lead is negligible due to the fact that Al is poorly soluble in lead. The concentrations of gold and metals from the PGM are small, but they may appear in the composition of the copper drosses.

These results were compared with the results from another study on PCB melting, but in iron slag, simulating the conditions of copper pyrometallurgy [37]. In both studies, phases/areas in the alloy after the process are noted, which are rich in copper (up to 95.7 wt.% Cu), bronze (up to 80.9 wt.% Cu, 37.3 wt.% Pb and up to 20.5 wt.% Sn), and lead (up to 85.4 wt.%). However, in [37], there were also noted phases very rich in iron, i.e., either Cu-Fe alloy (up to 44.4 wt.% Cu and up to 57.6 wt.% Fe) or pure iron (95.7 wt.% Fe). Thus, in the treatment of PCB scrap with copper metallurgy, iron contamination occurs, which is not the case in melting with lead. Moreover, in [37] a higher temperature was used, i.e., 1350 °C, which is necessary to melt the slag, while in this work, a lower temperature, of approx. 1110 °C, was used. These facts show that the end products obtained in both different processes are similar, but differ in technological conditions, of which melting of PCB scrap in lead seems to be the more advantageous technology.

## 4. Conclusions

The results of the research prove that it is possible to recycle non-ferrous metals, especially copper, tin, and others accompanying them (Ag, Au, PGM) from electronic scrap by melting it in lead and liquation. In lead, mainly copper and tin dissolve, forming bronze, and the presence of other metals results from their presence in the initial material, which was PCB scrap, and dissolution in lead. In relation to crude lead, the lead content in lead after melting drops to as much as 94%, and the content of other metals, mainly copper and tin, increases. As a result of melting PCBs in lead, there is no concentration with calcium and ferrous metals, and their concentration in lead is irrelevant from the point of view of liquation. In general, there is a relationship between the amount of pyrolysate used from the pyrolysis of PCB scrap and the amount of crude lead: the more pyrolysate is used, the more copper and tin will dissolve in the material. The best initial material in terms of copper and tin content in lead after the process, turned out to be the “new” pyrolysate. This was due to the fact that this pyrolysate had the most copper and tin, respectively, 9.745 wt.% and 1.42 wt.%, and for this reason, lead after melting also had the most of these metals, with the *m_PCB_*/*m_Pb_* = 0.1 g∙g^−1^, i.e., 3.206 and 1.358 wt.% of copper and tin, respectively. Overall, the higher the *m_PCB_*/*m_Pb_* ratio, the more lead and tin there are in the lead, to, respectively, 2.2056 and 0.8324 wt.%, for *m_PCB_*/*m_Pb_* = 0.1 g∙g^−1^.

Lead turned out to be as good a collector of non-ferrous metals as copper. The proposed method is an alternative to melting PCB scrap in copper, mainly due to the physicochemical properties of lead, such as low viscosity and lower heat of melting compared to copper. Melting PCB scrap in lead has many advantages, such as separation of scrap from aluminum, ease of liquation operation consisting only in cooling the top, and collecting copper drosses. It is postulated that the proposed method can be applied under conditions of lead pyrometallurgy. Copper drosses obtained as a result of liquation are copper concentrate from which copper and other metals, including precious and platinum group metals, can be easily recovered.

## Figures and Tables

**Table 1:** Chemical compositions (as oxides) of the initial materials after PCB scraps grinding and separation.

	Al_2_O_3_	SiO_2_	SO_3_	CaO	TiO_2_	MnO	Fe_2_O_3_	NiO	CuO	ZnO	Br	SnO_2_	BaO	PbO
	% by Mass													
“B1”	22	40.2	0.7	13.67	0.681	0.217	1.75	0.749	7.891	0.282	8	0.92	2.51	0.7
“B3”	51	32.0	0.5	7.83	0.556	0.026	0.97	0.589	2.040	0.124	-	0.94	2.85	0.2
“B5”	24	37.6	1.0	12.89	0.753	0.218	2.56	0.666	8.505	0.262	7	1.29	2.37	1.3
“server”	20	40.7	0.5	14.46	0.889	0.056	1.10	0.705	6.949	0.213	9	1.01	2.74	1.3
“new”	24	35.7	1.2	11.96	0.626	0.434	4.98	0.713	9.745	0.288	5	1.42	2.61	1.4

**Table 2:** Weight loss (∆*m*, %) resulting of pyrolysis of initial materials.

Type of PCB Material	% Weight Loss
“B1”	37.0
“B3”	82.7
“B5”	31.1
“server”	30.0
“new”	26.6

**Table 3:** Chemical compositions of the crude lead, averages and standard deviation of 10 analyses.

	Ca	Fe	Ni	Cu	Sn	Pb	Au	Other
% by mass	0.284	0.042	0.502	0.060	0.322	98.80	0.1	0.022
standard deviation	0.014	0.015	0.004	0.026	0.173	0.13	0.0	0.017

**Table 4:** Chemical compositions of the lead ingots after melting with PCB pyrolyzate “B1”, averages and standard deviation of 3 analyses.

*m_PCB_*/*m_Pb_* g∙g^−1^		Ca	Fe	Ni	Cu	Sn	Pb
0.0325	average, % by mass	0.311	0.127	0.589	0.772	0.447	97.8
	standard deviation	0.076	0.015	0.148	0.105	0.026	0.25
0.0650	average, % by mass	0.389	0.128	0.614	1.270	0.512	97.1
	standard deviation	0.079	0.012	0.053	0.082	0.090	0.19
0.1000	average, % by mass	0.376	0.133	0.632	1.830	0.643	96.4
	standard deviation	0.059	0.051	0.021	0.176	0.181	0.06

**Table 5:** Chemical compositions of the lead ingots after melting with PCB pyrolyzate “B3”, averages and standard deviation of 3 analyses.

*m_PCB_*/*m_Pb_* g∙g^−1^		Ca	Fe	Ni	Cu	Sn	Pb	Au
0.0325	average, % by mass	0.295	0.071	0.522	0.538	0.511	98.0	0.08
	standard deviation	0.051	0.030	0.007	0.040	0.054	0.15	-
0.0650	average, % by mass	0.274	0.160	0.675	1.050	0.624	97.2	0.09
	standard deviation	0.013	0.043	0.002	0.030	0.089	0.06	-
0.1000	average, % by mass	0.217	0.275	0.649	1.802	0.709	95.3	0.08
	standard deviation	0.043	0.095	0.020	0.060	0.019	0.55	-

**Table 6:** Chemical compositions of the lead ingots after melting with PCB pyrolyzate “B5”, averages and standard deviation of 3 analyses.

*m_PCB_*/*m_Pb_* g∙g^−1^		Ca	Fe	Ni	Cu	Sn	Pb	Au
0.0325	average, % by mass	0.267	0.154	0.667	0.955	0.429	97.5	0.04
	standard deviation	0.015	0.061	0.086	0.125	0.033	0.07	0.1
0.0650	average, % by mass	0.302	0.124	0.537	1.646	0.604	96.7	0.04
	standard deviation	0.037	0.083	0.052	0.049	0.054	0.30	0.1
0.1000	average, % by mass	0.368	0.094	0.631	1.800	0.852	96.2	0.05
	standard deviation	0.050	0.043	0.111	0.269	0.436	0.67	0.1

**Table 7:** Chemical compositions of the lead ingots after melting with PCB pyrolyzate “server”, averages and standard deviation of 3 analyses.

*m_PCB_*/*m_Pb_* g∙g^−1^		Ca	Fe	Ni	Cu	Sn	Pb
0.0325	average, % by mass	0.277	0.070	0.528	1.153	0.587	97.4
	standard deviation	0.011	0.045	0.005	0.093	0.200	0.35
0.0650	average, % by mass	0.350	0.118	0.546	1.935	0.558	96.4
	standard deviation	0.046	0.064	0.067	0.487	0.270	0.31
0.1000	average, % by mass	0.283	0.046	0.549	2.390	0.600	96.1
	standard deviation	0.013	0.006	0.033	0.334	0.260	0.49

**Table 8:** Chemical compositions of the lead ingots after melting with PCB pyrolyzate “new”, averages and standard deviation of 3 analyses.

*m_PCB_*/*m_Pb_* g∙g^−1^		Ca	Fe	Ni	Cu	Sn	Pb	Zn
0.0325	average, % by mass	0.275	0.059	0.532	1.225	0.821	97.3	0.030
	standard deviation	-	0.016	0.016	0.285	0.223	0.40	-
0.0650	average, % by mass	0.221	0.001	0.598	2.672	0.951	95.5	0.041
	standard deviation	0.072	0.028	0.029	0.560	0.436	1.02	0.034
0.1000	average, % by mass	0.290	0.076	0.539	3.206	1.358	94.4	0.097
	standard deviation	0.019	0.033	0.031	0.634	0.362	1.04	0.026

**Table 9:** Point chemical compositions of the lead ingots after melting with PCB pyrolyzate “new”, *m_PCB_*/*m_Pb_* = 0.1 g∙g^−1^, points of analysis from Figure 7.

No.	Al	Fe	Ni	Cu	Zn	Pd	Ag	Sn	Au	Pb
	% by Mass									
1	0.13	0.06	0.07	1.49	0.08	3.00	81.99	0.00	0.00	13.19
2	0.09	0.44	1.01	87.19	0.50	0.05	0.09	9.91	0.10	0.63
3	0.12	0.02	1.48	76.88	0.00	0.02	0.27	20.58	0.09	0.53
4	0.12	0.59	1.19	86.59	0.75	0.00	0.00	10.02	0.17	0.58
5	0.08	0.13	1.08	77.82	0.00	0.02	0.53	19.26	0.07	1.01
6	0.26	0.00	0.04	5.78	0.05	0.93	0.21	0.12	0.51	92.11

**Table 10:** Point and area chemical compositions of the lead ingots after melting with PCB pyrolyzate “B3”, *m_PCB_*/*m_Pb_* = 0.1 g∙g^−1^, points and area of analyses from Figure 8.

No.	Al	Fe	Ni	Cu	Zn	Pd	Ag	Sn	Au	Pb
	% by Mass									
1	0.18	0.17	74.20	0.00	0.14	23.74	0.13	1.45	0.18	0.17
2	0.13	1.08	69.93	0.00	0.00	26.04	0.13	2.62	0.13	1.08
3	0.22	0.02	0.41	0.00	0.30	0.00	0.18	98.87	0.22	0.02
4	22.98	0.16	0.47	0.37	0.53	0.00	0.00	75.48	22.98	0.16
5	0.22	0.00	0.37	0.00	0.34	0.00	0.22	98.86	0.22	0.00
6	0.28	0.01	0.08	0.00	1.20	0.00	0.30	98.14	0.28	0.01
7	0.18	0.17	74.20	0.00	0.14	23.74	0.13	1.45	0.18	0.17

**Table 11:** Point chemical compositions of the lead ingots after melting with PCB pyrolyzate “server”, *m_PCB_*/*m_Pb_* = 0.1 g∙g^−1^, points of analysis from Figure 9.

No.	Al	Fe	Ni	Cu	Zn	Pd	Ag	Sn	Pt	Pb
	% by Mass									
1	0.19	0.06	0.00	1.36	0.21	0.00	1.20	0.00	0.00	0.57
2	0.14	0.00	0.00	0.58	0.05	1.13	0.11	0.31	0.00	0.47
3	0.15	0.00	0.00	1.47	0.00	1.96	69.36	0.00	0.47	0.15
4	0.16	0.40	1.66	88.90	0.45	0.00	1.46	2.08	0.00	0.58
5	0.12	0.10	1.58	76.21	0.00	0.00	0.44	20.33	0.00	0.07

**Table 12:** Point chemical compositions of the lead ingots after melting with PCB pyrolyzate “new”, *m_PCB_*/*m_Pb_* = 0.1 g∙g^−1^, points of analysis from Figure 10.

No.	Al	Fe	Ni	Cu	Zn	Pd	Ag	Sn	Au	Pb
	% by Mass									
1	0.22	0.08	0.00	0.65	0.19	2.63	81.68	0.00	0.10	14.46
2	0.15	0.10	0.01	81.70	0.00	0.00	0.07	16.50	0.30	1.17
3	0.06	0.14	0.46	74.84	0.94	0.00	0.26	22.23	0.24	0.83
4	0.15	0.00	0.17	1.91	0.05	1.06	0.19	0.20	0.42	95.85
5	0.16	0.00	0.03	0.11	0.23	0.00	0.16	0.00	0.60	98.70

## References

[1] Sohaili J., Muniyandi S.K., Mohamad S.S. (2012). A Review on Printed Circuit Board Recycling Technology. Int. J. Sci. Eng. Res..

[2] Yu J., Williams E., Ju M. Review and prospects of recycling methods for waste printed circuit boards. Proceedings of the 2009 IEEE International Symposium on Sustainable Systems and Technology.

[3] Eswaraiah C., Kavitha T., Vidyasagar S., Narayanan S.S. (2008). Classification of metals and plastics from printed circuit boards (PCB) using air classifier. Chem. Eng. Process..

[4] Sanyal S., Ke Q., Zhang Y., Ngo T., Carrell J., Zhang H., Dai L.L. (2013). Understanding and optimizing delamination/recycling of printed circuit boards using a supercritical carbon dioxide process. J. Clean. Prod..

[5] Li J., Shrivastava P., Gao Z., Zhang H.-C. (2004). Printed Circuit Board Recycling: A State-of-the-Art Survey. IEEE Trans. Electron. Packag. Manuf..

[6] Tan Q., Liu L., Yu M., Li J. (2020). An innovative method of recycling metals in printed circuit board (PCB) using solutions from PCB production. J. Hazard. Mater..

[7] Wang H., Zhang S., Li B., Pan D., Wu Y., Zuo T. (2017). Recovery of waste printed circuit boards through pyrometallurgical processing: A review. Resour. Conserv. Recycl..

[8] Long L., Sun S., Zhong S., Dai W., Liu J., Song W. (2010). Using vacuum pyrolysis and mechanical processing for recycling waste printed circuit boards. J. Hazard. Mater..

[9] Qiu R., Lin M., Ruan J., Fu Y., Hu J., Deng M., Tang Y., Qiu R. (2020). Recovering full metallic resources from waste printed circuit boards: A refined review. J. Clean. Prod..

[10] Ma E., Prasad M.N.V., Vithanage M. (2019). Chapter 11-Recovery of Waste Printed Circuit Boards through Pyrometallurgy. Electronic Waste Management and Treatment Technology.

[11] Zeng X., Zheng L., Xie H., Lu B., Xia K., Chao K., Li W., Yang J., Lin S., Li J. (2012). Current Status and Future Perspective of Waste Printed Circuit Boards Recycling. Procedia Environ. Sci..

[12] Moskalyk R.R., Alfantazi A.M. (2003). Review of copper pyrometallurgical practice: Today and tomorrow. Miner. Eng..

[13] Rabah M.A. (1998). Combined hydro-pyrometallurgical method for the recovery of high lead/tin/bronze alloy from industrial scrap. Hydrometallurgy.

[14] Hoffmann J.E. (1992). Recovering precious metals from electronic scrap. JOM.

[15] Ghodrat M., Rhamdhani M.A., Brooks G., Rashidi M., Samali B. (2017). A thermodynamic-based life cycle assessment of precious metal recycling out of waste printed circuit board through secondary copper smelting. Environ. Dev..

[16] Peng Z., Li Z., Lin X., Tang H., Ye L., Ma Y., Rao M., Zhang Y., Li G., Jiang T. (2017). Pyrometallurgical Recovery of Platinum Group Metals from Spent Catalysts. JOM.

[17] Wood J., Creedy S., Matusewicz R., Reuter M. Secondary Copper Processing Using Outotec Ausmelt TSL Technology. Proceedings of the MetPlant 2011 Plant Design & Operating Strategies–World’s Best Practice.

[18] Gargul K. (2020). Ammonia leaching of slag from direct-to-blister copper smelting technology. AIMS Mater. Sci..

[19] Gupta G.S., Seetharaman S. (2014). Chapter 3-Metallurgical Production Technology. Treatise on Process Metallurgy.

[20] Bale C.W., Bélisle E., Chartrand P., Decterov S.A., Eriksson G., Gheribi A.E., Hack K., Jung I.H., Kang Y.B., Melançon J. (2016). FactSage Thermochemical Software and Databases-2010–2016. Calphad.

[21] Shishin D., Chen J., Jak E. (2020). Thermodynamic Modeling of the Pb-S and Cu-Pb-S Systems with Focus on Lead Refining Conditions. J. Phase Equilibria Diffus..

[22] Xu B., Guo X., Deng Y., Xiong H., Yang B., Liu D., Jiang W. (2018). Removal of Sulfur from Copper Dross Generated by Refining Lead. Miner. Met. Mater. Ser..

[23] Kern E.F. (1927). Method of Purifying Lead, Tin, and Lead-Tin Alloys.

[24] Cybulski A., Prajsnar R. (2013). Alternative method for copper removal from lead by application of aluminium. EMC.

[25] Friedrich B., Arnold A., Toubartz F. (2001). Cu-Pb-Me-S-balances during lead refining. EMC.

[26] Hallows R.L. (1940). Process for Refining Lead.

[27] Qiu K., Chen Q., Winkler P., Krger J. (2001). Behaviour of copper in refining of lead by fractional crystallization. Miner Process Extr. Metall..

[28] Chakrabarti D.J., Laughlin D.E. (1984). The Cu-Pb (Copper-Lead) System. Bull. Alloy Phase Diagr..

[29] Ellis T.W., Mirza A.H. (2010). The refining of secondary lead for use in advanced lead-acid batteries. J. Power Sources.

[30] McClincy R.J. (1966). The Influence of Additive Elements on the Decopperizing of Lead. Master’s Thesis.

[31] Ofte D. (1967). The viscosity of liquid uranium, gold and lead. J. Nucl. Mater..

[32] Assael M.J., Kalyva A.E., Antoniadis K.D., Banish R.M., Egry I., Wu J., Kaschnitz E., Wakeham W.A. (2010). Reference Data for the Density and Viscosity of Liquid Copper and Liquid Tin. J. Phys. Chem. Ref. Data.

[33] Roine A. (2002). Outokumpu HSC Chemistry for Windows, Version 5.1.

[34] Hanafi J., Jobiliong E., Christiani A., Soenarta D.C., Kurniawan J., Irawan J. (2012). Material Recovery and Characterization of PCB from Electronic Waste. Procedia Soc..

[35] Gutierrez-Perez V.H., Cruz-Ramirez A., Vargas-Ramirez M., Palacios-Beas E., Sanchez-Alvarado R.G. (2014). Silver removal from molten lead through zinc powder injection. Trans. Nonferrous Met. Soc. China.

[36] Karumb E.T., Taylor P.R. The Refining of Lead Bullion by Vacuum Distillation: A Literature Survey. https://mountainscholar.org/bitstream/handle/11124/170933/ETK_GRADS_2017_Poster.pdf?sequence=1.

[37] Wan X., Fellman J., Jokilaakso A., Klemettinen L., Marjakoski M. (2018). Behavior of Waste Printed Circuit Board (WPCB) Materials in the Copper Matte Smelting Process. Metals.

